# Histopathological evaluation of viscoelastic POSS administered subcutaneously using a rat model

**DOI:** 10.1016/j.toxrep.2025.102006

**Published:** 2025-03-22

**Authors:** Hamed Benghuzzi, Michelle Tucci, Drew Hildebrandt, Sukhendu Hait, Joseph Lichtenhan

**Affiliations:** a Jackson State University, 1400 John R. Lynch St, Jackson, MS 39217, USA; b University of Mississippi Medical Center, 2500 North State Street, Jackson, MS, USA; c Hybrid Plastics, 55 Wl Runnels Industrial Dr, Hattiesburg, MS 39401, USA

**Keywords:** Polyhedral oligomeric silsesquioxane, POSS, Histopathology, In Vivo Imaging Systems, IVIS®

## Abstract

**Introduction:**

Polyhedral oligomeric silsesquioxanes (POSS), have a structure resembling a cage in which the corners are silicon atoms bound with oxygen bridges. POSS cages may be dissolved in a polymer matrix or used as a cross-linker. When added into a polymer matrix, they remain non-toxic, improve mechanical properties, and resist biodegradation, which is important in tissue engineering. Limited studies evaluate long-term tissue responses to POSS gels.

**Methods:**

Adult male Sprague Dawley rat were used as a model to determine the tissue response towards multiple dosing of POSS gel compared to air injections for up to 180 days. A total of twenty-four rats was used for the study (Six rats were injected with the fluorescently labeled UVPOSS:POSS for imaging and eighteen additional rats were injected either with POSS SO1455 or air as a control group). Each group received three injections, and tissues were harvested 7, 14, and 21 days for early tissue responses, and 90- and 180-days post injection for late tissue reactions.

**Results:**

POSS vesicles formed after injection and remained pliable over 180 days. POSS complexed with Perylene dye could be imaged over 4 weeks showing lack of systemic mobility. Over the course of 180 days, POSS triggered a foreign body response with low- grade inflammation. The high viscosity nature of POSS resulted in the formation of a dense capsule preventing the material migration. **Conclusion**: POSS is encapsulated with connective tissue without long-term inflammatory cells or granuloma cell formation for 180 days in rats.

## Highlights

• POSS triggers a mimimal foreign body tissue response.
• The high viscosity nature of POSS prevents the material migration.
• Foreign body reaction surrounds the POSS similar in nature to that observed with silicon.

## Graphical Abstract

**Figure:**
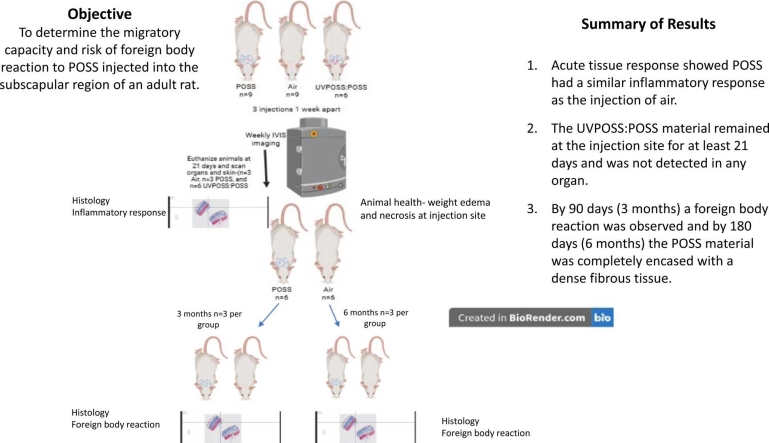

## 1 Introduction

Dermal fillers are a cosmetic dermatology procedure that are used to treat superficial and deep wrinkle lines, scars, and other signs of aging. They are injected into the skin to create volume and restore fullness of the tissue. An ideal injectable gel for biomedical applications should be easily injectable, volume-stable, nonantigenic, and non-migratory. All injectable fillers are polymeric biomaterials that are classified into temporary ( Hyaluronic acid < 1 year), semipermanent (poly-d-lactic acid or calcium hydroxylapatite <2 years), or permanent (polymethylmethacrylate (PMMA) and silicon). Permanent fillers like PMMA seem appealing because they last longer than other types of fillers; however, these fillers can consist of larger particle sizes that can form lumps under the skin. Silicone based,fillers seem appealing because they last longer than other types of fillers and are more pliable than PMMA. Silicone injectables have been used clinically in liquid and gel forms. For example, silicone is Food and Drug Administration (FDA) approved for use as a medical-grade injectable for treating retinal detachment in ophthalmology [1]. It helps hold the retina in place during vitrectomy surgery [1], and a second procedure is needed to remove the silicone. However, silicone injectables are often used off label for cosmetic purposes, such as plumping lips, reversing acne scars, and filling under-eye hollows, and silicone containing materials’ safety and efficacy in these procedures remains controversial [2], [3], [4], [5], [6], [7].

Unlike temporary fillers, injected silicone is permanent and does not break down in the body, thus increasing the risk for the development of a foreign body reaction (FBR). The reaction occurs as the cells within the immune system become attracted to the foreign material (silicone) and attempt to degrade it. If this degradation fails, fibroblasts encase the material producing a fibrotic tissue barrier which shields it from the body.

**Diagram 1:**
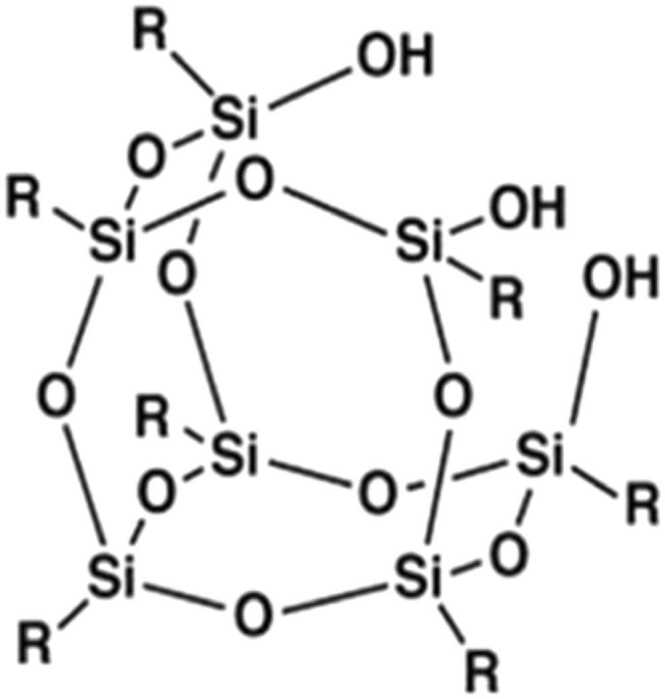
Structure of POSS SO1455-Hybrid Plastics, Inc. Hattiesburg, MS.

A newer polymeric gel known as polyhedral oligomeric silsesquioxane (POSS), comprised of iso-octyl groups is an organic-inorganic hybrid compound containing silicone (Si) atoms linked to chemically modified organic moieties that has gained attention as a hemostatic agent for use in traumatic bleeding and epistaxis. POSS molecules adopt a cage-like or polymeric structures with Si-O-Si linkages and tetrahedral Si vertices (Diagram 1). POSS has desirable physical properties such as optical clarity, tissue adhesion, and viscoelasticity and is a promising gel-device for treatment of noncompressible traumatic bleeding injuries. [8] POSS gel is not intended to be left within the body; however, the possibility exists. Currently, there are no studies to determine the migratory capacity and the risk of foreign body reactions to POSS that remains for a prolonged periods within the body.

The overall objectives of this study were to investigate movement of POSS from site of injection and the body’s reaction to POSS by evaluating histopathological changes associated with repeated dosing and long-term chronic exposure of POSS gel in a rat model. We hypothesized that POSS gel left within the body would be encapsulated and remain at the injection site.

## 2 Materials and methods

### 2.1 Test articles

Three test substances were employed in this study: (a) 100 % POSS polymer gel, (b) 90 % POSS gel and 10 % of a UV fluorescent POSS (UVPOSS:POSS), and (c) air. The POSS and UVPOSS: POSS gels [9] were manufactured by *Hybrid Plastics Inc.* under the trade name POSS® (product number of SO1455 and aminopropyl-isobutyl-POSS). FDA approval for POSS gel is being considered for use as a conformable hemostatic epistaxis device under the trade name Statbond™.

### 2.2 Synthesis of UVPOSS:POSS gel (POSS-Perylene bisimide)

The synthesis of UVPOSS was carried out according to the method reported by Tucci et al. [9]. Briefly, a mixture of perylene dianhydride (90 mg) was mixed with 0.5 g aminopropyl-isobutyl-POSS (Hybrid plastics, Hattiesburg, MS) and refluxed for 48 hours with N-methyl-2-pyrrolidone (NMP). The solvent was removed by distillation and the resultant product was a red residue. UVPOSS (10 %) was mixed with POSS(SO1455) (90 %) for the resultant UVPOSS:POSS material.

### 2.3 Matrix of substances

The matrix of substances injected is provided in Table 1. Air was utilized as control for the purpose of determining the background biological and tissue inflammatory response to the physical injection and air pocket. The POSS gel polymer was of primary interest for elucidating the biological response of the device under chronic exposure conditions. UVPOSS is fluorescently active and is easily recognized using the IVIS® technique. POSS:UVPOSS contains 10 % UVPOSS and 90 % POSS SO1455 gel [9] by weight. The POSS material is 100 % POSS SO1455 gel. In order to further test the shape retention or migration of the gel, a “streak” of POSS was injected subcutaneously and observed for 7 days.

**Table 1:** Injection substances for chronic tissue studies in rats.

**Injected Material**	**Purpose**
**POSS SO1455**	For assessment of biological response of gel polymer
**POSS:UVPOSS**	For ease of in vivo detection relative to migration
**Air (Control)**	Control for air-surface interface to establish baseline tissue response.

### 2.4 Experimental design

Following protocol approval from the University of Mississippi Medical Center Institutional Animal Care and Use Committee (IACUC) protocol #1518, twenty-four Sprague Dawley adult male rats were randomly divided into three groups: UVPOSS:POSS (n = 6), POSS (n = 9) and Air (n = 9). Under isoflurane anesthesia, the hair on the back of the neck between the shoulder blades was shaved. The first injection was placed on the upper right side, the second injection a week later was placed on the upper left side and the third injection a week later was placed in the mid-region between the bottom portion of the shoulder blades. Each animal received an injection weekly for three weeks (Table 2). Under isoflurane anesthesia, fifty microliters of each test article were administered subcutaneously (SC) placed as described above, using an insulin syringe with a 25- gauge needle. Twenty-one days after the first injection, three animals per group were euthanized and tissues at the injection site along with vital and reproductive organs were harvested to determine migration of the material. The remaining animals’ body weights and injection sites were evaluated weekly, and at 90 days three animals in the POSS and air groups were euthanized and injection sites were harvested for histopathological assessments. At 180 days the remaining animals in each group were euthanized and injection site and vital and reproductive tissues were harvested. The animals in the UVPOSS: POSS group were evaluated using IVIS® weekly to determine migration of the UVPOSS:POSS material. The organs collected from the UVPOSS:POSS group at 21 and 180 days were analyzed using IVIS® to determine migration of the materials from the injection site.

**Table 2:** Detailed experimental design for all groups. Animals were injected subcutaneously and euthanized following standard lab protocols.

Day	Procedures	UVPOSS:POSS (Longitudinal imaging-only), N = 6`	POSS N = 9	Air (control) N = 9
1	Injection #1	6 animals injected	9 animals injected	9 animals injected
7	Injection #2	6 animals injected	9 animals injected	9 animals injected
14	Injection #3	6 animals injected	9 animals injected	9 animals injected
21	Euthanize	3 animals euthanized	3 animals euthanized	3 animals euthanized
90	3 months	0 (Longitudinal imaging)	3 animals euthanized	3 animals euthanized
180	6 months	3 animals euthanized	3 animals euthanized	3 animals euthanized

### 2.5 In vivo imaging systems (IVIS® imaging)

Under isoflurane anesthesia, animals receiving UV:POSS, IVIS® imaging was performed immediately and 3 hours after injection to assess the signal intensity. Animals then were imaged weekly until the dye was undetectable. The animals were weighed weekly and imaged under the IVIS® Spectrum in fluorescence mode (Perkin-Elmer, USA) to visualize the fluorescent POSS at excitation/emission of 535/660 nm, auto exposure (system automatically adjusted the exposure level based on the detected signal level) and small binning (binning refers to the combining adjacent pixel on the CCD sensor to improve sensitivity). Small binning was selected so fewer pixels are combined which results in higher resolution. Regions of interest (ROIs) were drawn over the fluorescent areas, and average radiant efficiency indicating fluorescence intensity was used as an indicator UVPOSS:POSS location within the animal.

### 2.6 Organ and skin ex vivo IVIS® imaging

Six animals were in the UVPOSS:POSS group for longitudinal imaging with half of the animals euthanized after 21 days and remaining animals at 180 days by an overdose of isoflurane. The vital organs (liver, kidney, heart, spleen, lung, and adrenal glands), reproductive organs (testes, seminal vesicles, prostate, and skin at the site of injections were harvested and imaged using IVIS® to check for whole organ fluorescence.

### 2.7 Histology and histological assessment

The tissues at the interface of the injection sites and naive tissue were taken and fixed in 10 % formalin. The fixed tissues were embedded in paraffin and sectioned at 5 µm. The sections were stained with either hematoxylin/eosin (H&E) or Masson’s trichrome stain. The slides were dried and digitized using Philips’s slide scanner. Cross-sections were divided into naïve tissue, injection site edge, and injection site center. For each section, four random visual fields were chosen for histological assessments. Histologic assessments included extravasation of inflammatory cells around POSS droplets, the number of giant cells, and areas of fibrosis. We used a double-blind method with two experienced examiners for each specimen to ensure that there was an agreement regarding the FBR to POSS injections. An additional blinded examiner reviewed all findings; any differences in evaluations between the two examiners were addressed by consensus.

### 2.8 Statistical analysis

The numerical data are expressed as mean ± SD. SigmaPlot 12 software (San Jose, CA, USA) was used to determine statistical significance. One-way analysis of variance for repeated measures followed by Dunnett’s post-hoc text were used to evaluate changes over time within a particular group. Between-group comparisons were assessed with the Student T-test. A *p* < 0.05 was considered significant.

## 3 Results

### 3.1 Systemic health effects

**Fig. 1:**
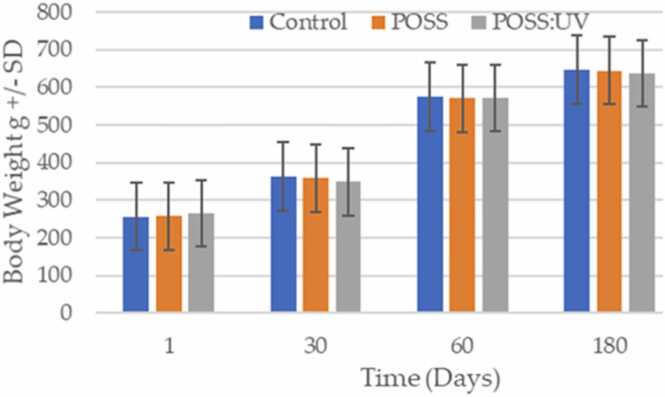
Body weights for experimental groups for the duration of the experiment. There was no significant difference between groups.

Each animal in this study was attended daily by a veterinary care team. Over the 180-day study period it was notable that injections of the POSS gel, POSS:UVPOSS dye, or air (control) did not elicit any pain, pyrogenicity, or changes in animal behavior. The animals gained the expected dietary weight (Fig. 1), and maintained normal activity. No animals were euthanized prior to outcome dates.

### 3.2 Anatomical findings

**Fig. 2:**
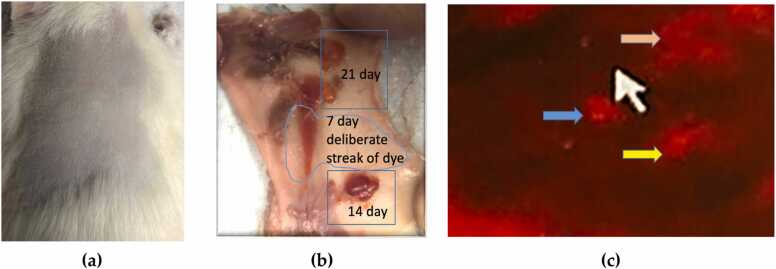
(a) Representative subcutaneous rat tissue injection sites for the UVPOSS after 7, 14, and 21 days. Note the orange-red vesicle color results from the fluorescent UVPOSS gel. Injections of the POSS gel alone resulted in translucent vesicles. (b) Normal appearance of muscle in contact with POSS. (c) Injection site IVIS® imaging, white arrow shows tissue background and blue arrow represents UVPOSS:POSS after 7 days( injection #3), yellow arrow represents location of UVPOSS:POSS 14 days (injection # 2) and orange arrow represent UVPOSS:POSS at 21 days (initial injection).

It is worth mentioning that test substances (see Table 2) injected subcutaneously in 50 µL amounts to the mid-scapular region of the rat revealed significant findings. Upon injection, POSS SO1455 gel and POSS:UVPOSS formed soft pliable vesicles between the dermal layer and muscle tissue. The air control injection did not form vesicles. Each of the three injection sites could be seen easily at necropsy. The shape of the POSS vesicles was somewhat oblong (drop-like). This shape is common to the size of droplet delivered by a 25 gauge needle. The vesicle formation was attributed to the hydrophobic nature of the gel. IVIS® imaging showed each location of the three POSS:UVPOSS injections (Fig. 2 C) as well as a decline in signal intensity over time. Complete signal loss occurred by 28 days post injection.

Fig. 2 shows the area of injection and the necropsy site indicating streaked 7-day, normal 14-day, and 21-day injection sites for UVPOSS:POSS. The lack of migration, and resilience of the gel, are attributable to its high viscosity and adhesive qualities.

**Fig. 3:**
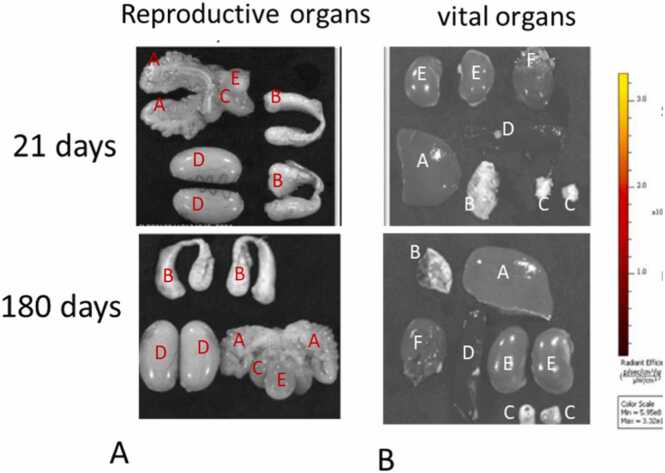
**A and B.** Representative IVIS® imaging of the (A) reproductive organs (A: seminal vesicles, B: epididymis, C: prostate, D: Testes, E: Bladder) and (B) vital organs (A: liver, B: lung, C: adrenal gland, D: spleen, E: kidney, and F: heart) of male rats with subcutaneous injections of UVPOSS:POSS at 21 and 180 days.

Organs were harvested and UVPOSS:POSS migration was evaluated at 21 days and 180 days by IVIS® imaging (Fig. 3 A and B). No detection of UV signal was seen above background in the vital organs (kidneys, spleen, adrenals heart, lung, or liver) or reproductive organs (testes, seminal vesicles, epididymis, and prostate), further reinforcing the IVIS findings of POSS migration away from the injection sites.

### 3.3 Biological tissue response

Tissues at the injection site were harvested at necropsy and processed for histopathology to evaluate tissue responses at 7, 14, 21, 90, and 180 days.

### 3.4 7-day histopathology

**Fig. 4:**
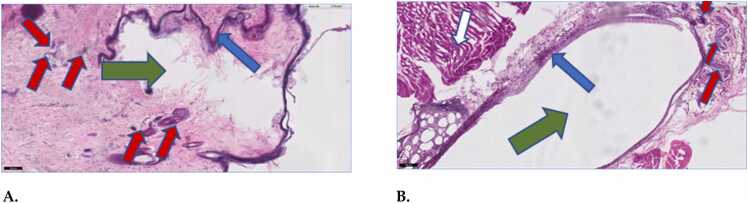
**A and B.** Tissue histopathology (digitized 5x, H&E)) after 7- day exposure to (A) injected air (air pocket in photo) 10x and (B) POSS 5x. The green arrows represent the disruption of the tissue by the injection of (A) air and (B) POSS. The blue arrows show the immediate response of a thin fibrous tissue, and red arrows show the presence of macrophage cell types. The white arrow represents muscle.

Tissue exposed to the air pocket and the POSS gel for 7 days are shown in Fig. 4. At the microscopic level, inflammatory cells (mainly macrophages and giant cells) are detected for both the air and UVPOSS:POSS (dye) exposed tissues. Comparatively, more inflammatory cells were present around the UVPOSS than around the air injection pocket. However, it should be noted, in both cases, that inflammatory cells are narrowly localized near the edges of each injection pocket. One-two layers of macrophages surround the gel, and fibroblast are present, which is similar to the tissue response around the control air pocket. POSS exhibited an initial biological inflammatory response which consisted of the presence of inflammatory macrophage cells. Although macrophage size in the POSS-treated tissues appeared larger, the number of cells counted in ten fields of view were not statistically different.

### 3.5 14-day histopathology

**Fig. 5:**
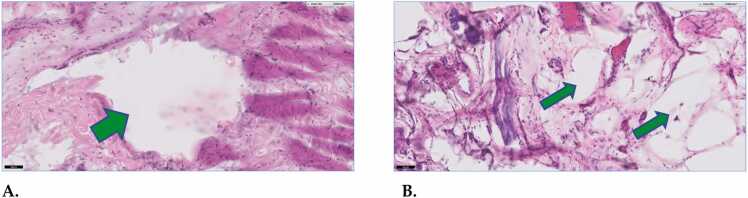
Tissue histopathology (digitized 20 x, H&E)) after 14-day exposure to (A) injected air (air pocket in photo) and (B) the POSS (POSS pocket in photo). The green arrow represents the pocket.

The histopathology images shown in Fig. 5 represent the 14-day tissue response to the air-injection pocket and the POSS. Histopathology images were focused on the tissue-air and tissue-POSS interface, and neither treatment showed abnormal tissue reactions. At the microscopic level, few inflammatory cells were detected near the air pocket in the control group and few inflammatory cells were observed near the control group or near POSS surface in the test groups. In both cases, there was an absence of necrosis with no evidence of an abnormal fibrotic response.

### 3.6 21-day histopathology

**Fig. 6:**
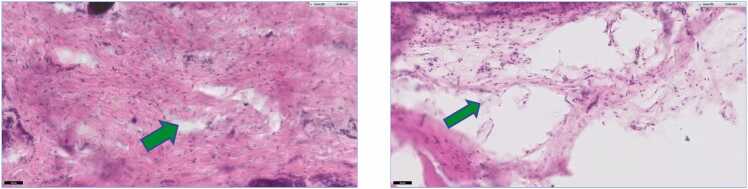
Tissue histopathology after 21-day exposure (20)x to (A) injected air and (B) the POSS. The green arrow represents the location of the 21-day injection.

The images in Fig. 6 represent the 21-day biological response after injection of air or the POSS gel. At the microscopic level few inflammatory cells were detected for either of the injected materials (less than 10 cells in 4 fields per view). In both cases a pocket where the air or POSS was injected was still visible, and fibroblasts were the main cell components within tissues. In the case of the POSS gel, the pocket was larger and surrounded by loose collagen, and POSS was see within the tissue.

### 3.7 90-day histopathology

**Fig. 7:**
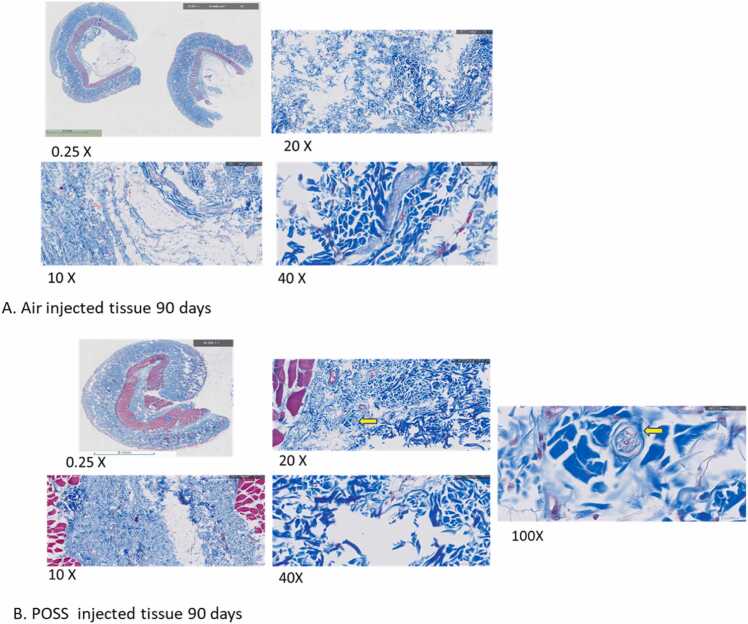
**A and B**. Representative cross sections stained with Masson Trichrome after 180-day exposure to (a) injected air and (b) POSS. The yellow arrow on 20x image of POSS injected with air depicts the presence of encapsulated POSS. The image was enlarged to 100X to show the encapsulated POSS (yellow arrow).

Similar tissue composed of fibroblast, collagen deposits, and young capillaries along with sebaceous glands and hair follicles was observed in the experimental groups. The trichrome stained images in Fig. 7A and B represent the 90-day biological response after injection of air or POSS gel. The POSS gel still was detected in individual pockets (yellow arrow on Image 7B). There was an increase in fibroblastic activity around the air and POSS pockets. At the 90 days phase acute inflammatory cells were lacking and there did not appear to be any adverse response to the POSS material.

### 3.8 180-day histopathology

**Fig. 8:**
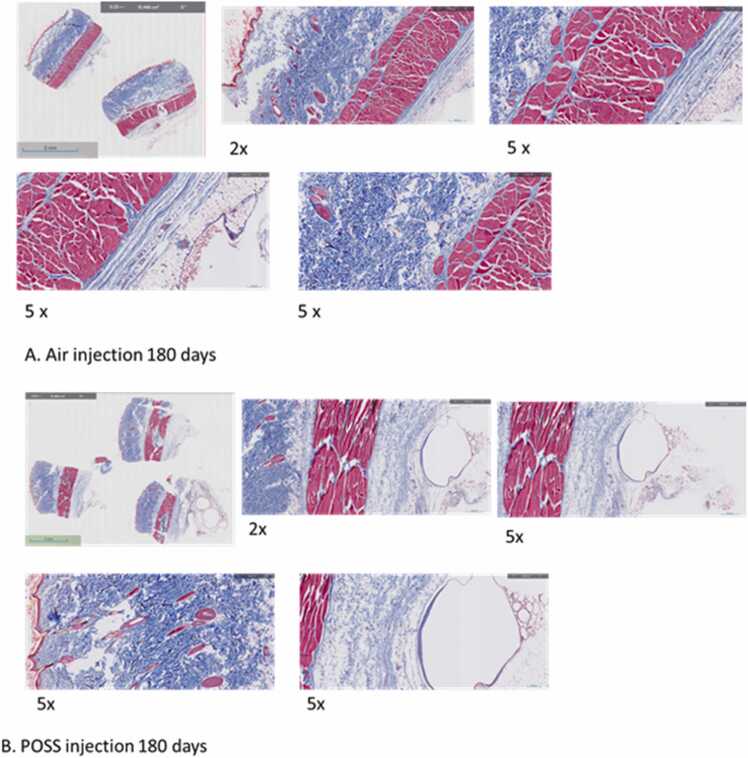
**A and B.** Represented Masson Trichrome tissue histopathology after 180-day exposure to (a) injected air and (b) the POSS.

Similar tissue composed of fibroblast, collagen deposits, and young capillaries along with sebaceous glands and hair follicles was observed in the experimental groups. The images in Fig. 8A and B represent the 180-day biological response after injection of (A) or the POSS gel (B). The tissues harvested from animals in the air group were unremarkable and without evidence of inflammatory cell types. No untoward responses were observed, nor is there a visible location where the air pockets were established. The loose connective tissue was measured from the muscle to the subcutaneous tissue in both groups (Table 3). The loose connective tissue was approximately 3x greater in the POSS gel (B); note Masson trichrome stains the muscle red, and connective tissue blue (Table 3 (p < 0.001). In Fig. 8B, the muscle tissue harvested from animals receiving POSS gel injections appeared normal without evidence of POSS within the muscle tissues. Disordered loose connective tissue is presence adjacent to the muscle tissue in the POSS-injected tissues.

**Table 3:** Measurement of loose connective and vesicles.

Group	Loose connective mm ± SD	Vesicle area mm^2^
Air	1.4 ± 0.55	None noted
POSS	4.08 ± 0.20*	2.71 ± 1.04

* p < 0.001

Interestingly, the retrieved tissues from the POSS experimental groups showed evidence of similar- sized POSS vesicles. The area of the vesicles measured between 1.17 mm^2^ and 3.9 mm^2^ with an average vesicle area of 2.71 mm^2^ ± 1.04 mm^2^. In four high-powered fields of view approximately 20 acute inflammatory cells (macrophages) were counted in one retrieved tissue, while the other tissues in the POSS experimental groups were lacking acute inflammatory cells. A classical foreign body reaction was observed around the POSS vesicles which consisted of encasing the vesicle with dense fibrosis tissue. The tissue thickness encasing the vesicles ranged between 97.89 µm and 13.09 µm with an average thickness of 38.16 µm ± 22.16 µm.

Over the 180-day study period we observed the POSS only within the subcutaneous pocket. It did not spread to adjacent muscle, nor into the body cavity or organs. Upon necropsy POSS vesicles were still evident at the site.

## 4 Discussion

Cage-like silsequioxanes have interesting properties that distinguish them from conventional oligo- and polymeric systems. They are distinguished by their nanometer size, which determines their use as additives to polymeric materials, composites, biomaterials, etc. Polyhedral oligomeric POSS used in this study is a cage-like silsesquioxane organic–inorganic hybrid material [10], [11], [12]. Its structural formula is (RSiO_1.5_)n (n ≥ 4), and its nanostructures have diameters ranging from 1 to 3 nm [13], [14], [15]. The presence of three SiO bonds together imparts superior mechanical, thermal, and chemical stability [16], [17]. The stability of POSS was evident as it was seen in the same location for the duration of the study. The histopathological observations and IVIS® imaging data allow us to confirm the POSS gel does not migrate from the injection site within the first 21 days. According to the literature, there are 2 types of migration that can be seen with injections of silicone. If the sizes of the droplets have a large-volume, then silicone will migrate along tissue planes and become encased, while small-volume silicone will undergo phagocytosis by macrophages. Small-volume silicone droplets of < 15 µm will be transported through lymphatic vessels. In our study, the data suggests that injections of large- volume POSS material does not migrate from the injection site and behaves similar to large-volume silicone. This observation is supported by IVIS® imaging over 21days and also by the presence of the vesicles in the same location after 6 months. The finding that no intensity readings above zero were found in the organ level for the UVPOSS:POSS gel obtained from IVIS® (Fig. 3) suggest that elimination via a biological process did not occur during the 21- day exposure period. It should be noted that we found that the region of interest (ROI) analysis of the material showed a decrease in radiant efficiency over 21 days, and by 28 days the intensity of the signal was diminished [9] which prevented us from continuing longitudinal measurements out 180 days.

Several studies have shown that POSS can be used as a component in the formation of biomedical devices which are non-toxic and show high biocompatibility [18], [19], [20]. For example, Zhang and colleagues prepared biodegradable hybrid double-network (DN) hydrogels using a covalent binding strategy between natural biomacromolecules (chitosan and gelatin) and inorganic POSS units to support osteogenesis [21]. The resulting device was a porous structure in which POSS was used to enhance the mechanical properties and biodegradability. The POSS-hydrogel device was tested *in vitro* to specifically evaluate the proliferation and differentiation of mesenchymal stem cells (MSCs) towards osteoblasts [21]. They further tested the material to heal a critical-sized defect by placing the hybrid hydrogel containing MSC into the skull of a rat. The results showed POSS-incorporated material can significantly promote *in situ* bone regeneration and mineral apposition. Our study identified the tissue reactions associated with long-term exposure to POSS. Overall, the body’s reaction to POSS is similar to what is reported for silicone. It should be noted that the form and topography of the surface of the biomaterial determines the composition of the foreign body reaction (FBR). With biocompatible materials, the composition of FBR at the implant site is controlled by the surface properties of the biomaterial, the form of the implant, and the relationship between the surface area of the biomaterial and the volume of the implant. For example, high surface-to-volume implants such as fabrics or porous materials will have higher ratios of macrophages and often form foreign body granuloma cells (FBGCs) in the implant site while smooth-surface implants tend to be associated with significant development of fibrosis tissue encasing the implant. Generally, with smooth surfaces, fibrosis (i.e., fibrous encapsulation) surrounds the biomaterial, isolating the implant and from the local tissue environment. In some cases, the FBR can consist of macrophages and/or foreign body granuloma cells at the tissue–implant interface for the lifetime of the expsoure [22], [23], [24], [25], [26], [27], [28], [29], [30].

Normally, the formation of the FBR starts similar to a physiological healing response following exposure of the foreign material [30], [31] and can be divided into overlapping stages, similar to the phases which define normal wound healing such as: (1) protein adsorption and formation of provisional ECM, (2) acute inflammation, and (3) chronic inflammation [32]. In our study, we found similar reactions within the first 21 days when either air or POSS was injected into the tissue. Even with multiple injections of the POSS material, the inflammatory phase was short lived. By 90 days the FBR was forming and by 180 days post injection, the material was completely encased with a dense fibrous tissue.

Previous studies in the literature show that implanted smooth silicone implants had reduced inflammatory reactions as well as reduced physical stimulation [10], [11]. Although smooth implants have been used consistently as implantable devices for breast reconstruction, their frequency of use is decreasing because the incidence of capsular contracture [31]. The smooth surface of POSS also generated a condition with low inflammatory cell types, as well as producing a dense fibrous capsule encasing the material by 180 days.

In addition to the smooth surface, the size of the material can also guide the fibrous capsule. In a study of liquid silicone intradermal injection into rat skin [32], the FBR monitored over 10 weeks increased in conjunction with the size of injected droplet. Interestingly, the numbers of inflammatory cells per higher power field were statistically higher in the first 4 weeks after injection comparable to studies of tissue responses toward large volume injections of silicone [35], [36].

In numerous studies where liquid silicone was injected as filler, the estimated frequency of granuloma formation was 1–10,000 [33], [34]. Similar to our study, injection of liquid silicone can cause the presence of large vacuoles and early presences of inflammatory cell types [35], [36].

Cakmak et al., estimated that rats age about 35 times faster than humans [32], and in their study with different-sized silicone droplets injected subdermally, there was a normal FBR response estimated to be between 3 and 7 years [32]. In our study, using the POSS injections, we also show a normal FBR response without the presence of foreign body granuloma cells estimated to be at the equivalent of 3, 8, and 17.5 human years based upon the rat age estimations. It should be noted that the POSS injections did not form firm nodules that would indicate capsular constriction. The nodules were pliable upon recovery at each time point. This suggests that POSS safety may be similar to a large-volume silicone implants.

Overall, POSS injection triggers a foreign body response with minimal tissue reactivity and low- grade inflammation. POSS lattice structure is not broken down in the injection site and the high viscosity nature of the material forming a capsule prevents the material from migration.

## 5 Conclusions

The purpose of this long-term exposure study was to gain more insight into the foreign body response towards POSS gel. The findings support measurable formation of a dense FBR that allows for gel to be encapsulated and remains as a soft-pliable vesicle at the site of injection.

### 5.1 Translational relevance

Synthetic materials induce to varying extents an immune-mediated FBR, which leads to formation of a capsule of dense fibrous tissue which surrounds the material. In this experimental design, we also found that a normal FBR response following multiple injections of the POSS material. After 21 days, there was minimal presence of neutrophil and macrophage cells found in the tissues surrounding the injected material, and the material was fully encapsulated and remained in the same location within these vesicles for 6 months.

### 5.2 Limitations

Conjugation of perylene to POSS resulted in fluorescence shifts that were no longer detectable in the IVIS® fluorescent range by 28 days. Perylene dye is an indicator dye for ammonia; ammonia perhaps a better choice would have been a stable dye such as BODipy. Furthermore, additional animals for the collection and analysis of the POSS material from the vesicles after wound harvest would have provided a more robust analysis of the concentration and molecular signature remaining at the site. Finally, perfusion of additional animals during euthanasia with Microfil™ would have allowed for precise quantitative estimations of the tissue volume by MicroCT analysis along with estimations of capillary density differences in the groups.

## Funding

Department of Defense Award Hemostatic Agent Development: W81XWH-17-C-0184-Subaward , University of Mississippi Medical Center.

## Authors

HB, MT, and DH have no financial or conflicts of interest. JL holds patents for POSS and both JL and SH are affiliated with and have financial interest in the company that designs/manufactures/sells the material used in this study. HB, MT, DH, JL all have contributed to the design and implementation of the experiments. MT, HB, and DH were involved with the majority of writing the manuscript. JL was involved with editing and reviewing the manuscript. SH was involved with design and construction of the UVPOSS:POSS material.

## Institutional Approval

University of Mississippi Medical Center, IACUC protocol #1518 approval was obtained prior to conducting experiments. United States Department of Defense also evaluated the protocols prior to initiating the study.

## Disclaimer/Publisher’s Note

The statements, opinions and data contained in all publications are solely those of the individual author(s) and contributor(s) and not of MDPI and/or the editor(s). MDPI and/or the editor(s) disclaim responsibility for any injury to people or property resulting from any ideas, methods, instructions or products referred to in the content.

## CRediT authorship contribution statement

**Lichtenhan Joseph:** Funding acquisition, Conceptualization. **Hait Sukhendu:** Resources, Methodology. **Hildebrandt Drew:** Writing – review & editing, Methodology, Investigation, Data curation, Conceptualization. **Benghuzzi Hamed:** Writing – review & editing, Methodology, Investigation, Formal analysis. **Tucci Michelle:** Writing – original draft, Methodology, Investigation, Formal analysis.

## Declaration of Competing Interest

The authors declare the following financial interests/personal relationships which may be considered as potential competing interests: Joseph Lichtenhan reports financial support was provided by US Department of Defense. Joseph Lichtenhan reports a relationship with Hybrid Plastics, Inc LLC that includes: employment and funding grants. Sukendu Hait reports a relationship with Hybrid Plastics that includes: employment. Joseph Lichtenhan has patent issued to Joseph Lichtenhan. If there are other authors, they declare that they have no known competing financial interests or personal relationships that could have appeared to influence the work reported in this paper.

## Data Availability

Data will be made available on request.
